# Enzymatic Modifications of Chitin, Chitosan, and Chitooligosaccharides

**DOI:** 10.3389/fbioe.2019.00243

**Published:** 2019-09-27

**Authors:** Michal Benedykt Kaczmarek, Katarzyna Struszczyk-Swita, Xingkang Li, Miroslawa Szczęsna-Antczak, Maurycy Daroch

**Affiliations:** ^1^Institute of Technical Biochemistry, Lodz University of Technology, Łódź, Poland; ^2^School of Environment and Energy, Peking University Shenzhen Graduate School, Shenzhen, China

**Keywords:** chitin, chitosan, chitooligosaccharides, enzymatic modifications, lytic polysaccharide monooxygenase, chitin deacetylase, chitinase, chitosanase

## Abstract

Chitin and its N-deacetylated derivative chitosan are two biological polymers that have found numerous applications in recent years, but their further deployment suffers from limitations in obtaining a defined structure of the polymers using traditional conversion methods. The disadvantages of the currently used industrial methods of chitosan manufacturing and the increasing demand for a broad range of novel chitosan oligosaccharides (COS) with a fully defined architecture increase interest in chitin and chitosan-modifying enzymes. Enzymes such as chitinases, chitosanases, chitin deacetylases, and recently discovered lytic polysaccharide monooxygenases had attracted considerable interest in recent years. These proteins are already useful tools toward the biotechnological transformation of chitin into chitosan and chitooligosaccharides, especially when a controlled non-degradative and well-defined process is required. This review describes traditional and novel enzymatic methods of modification of chitin and its derivatives. Recent advances in chitin processing, discovery of increasing number of new, well-characterized enzymes and development of genetic engineering methods result in rapid expansion of the field. Enzymatic modification of chitin and chitosan may soon become competitive to conventional conversion methods.

## Introduction

The interest in natural polymers has increased substantially over the last three decades. Chitin, the second most abundant, after cellulose, biopolymer on earth can be obtained from many sources including marine crustacean shell-waste material, insects, and exoskeleton of invertebrates. The worldwide market for Chitosan Derivatives is expected to grow at a CAGR (Compound Annual Growth Rate) of roughly 6.3% over the next 5 years, will reach 53 million USD in 2024, from 36 million USD in 2019 (Global Info Research, 2019), driven by the growing investments in new drug development, emerging biomedical applications and expanding non-medical uses such as detoxification of water and wastewater. There is also an increased interest in organic farming and certified use of biodegradable chitosan products as fertilizers (Global Industry Analysts Inc., 2016). Research on these biopolymers focuses, among other issues, on the search for new and improved production methods. Key areas of interest include: enzymatic conversion (Roberts, 2008), chemical, or physical modifications of polysaccharides to extend their applicability (Zhao et al., 2013) exploration of the mechanisms of biological activity of the said polymers and products of their physical, chemical or enzymatic degradation, and the biochemical and molecular characterization of chitosanolytic and chitinolytic enzymes synthesized by numerous organisms (Duo-Chuan, 2006; García-Fraga et al., 2015).

Biotransformation of chitin into chitosan through enzymatic deacetylation can be achieved with chitin deacetylases (EC 3.5.1.41, ChDa). This enzymatic reaction has several advantages over the traditional chemical processes, most importantly, the production of chitosan with higher molecular weight and the desired degree of deacetylation (Tsigos et al., 2000). Other enzymes involved in chitin and chitosan conversion are chitinases (EC 3.2.1.14) and chitosanases (EC 3.2.1.132). Both of them catalyze the hydrolysis of glycosidic bonds but differ in substrate specificity, hydrolysing bonds of chitin and chitosan, respectively (Jaworska, 2012). Obtained chitooligosaccharides can be further enzymatically modified by chitooligosaccharides deacetylases (EC 3.5.1.105, CODa) to obtain products with desired chain arrangement (Hirano et al., 2017). In recent years, lytic polysaccharide monooxygenases (LPMOs) (EC 1.14.99.53-56) attracted the attention of scientists. Generally, these enzymes are capable of cleaving glycolic bond in crystalline forms of polysaccharides through oxidizing either C1 or C4 of the glucopyranose ring. Chitin-active LPMO was first demonstrated in 2010 for the *Serratia marcescens* AA10 (CBP21) (Vaaje-Kolstad et al., 2010). In contrast to glycoside hydrolases (GHs), such as chitinases and chitosanases, LPMOs are capable of directly cleaving glycolic bonds in highly crystallized chitin (Mutahir et al., 2018). To date, LPMOs with chitinolytic activities have been identified in carbohydrate auxiliary activity families AA10, AA11, and AA15 (Hemsworth et al., 2015). Currently, all these enzymes are increasingly seen as useful tools toward biotechnological production of chitosan and chitooligosaccharides (COS), especially when a controlled, non-degradative, and well-defined process is required (Hamer et al., 2015). The main limitation of enzymatic methods lies in the multi-step character of this process and high costs of enzyme production, which makes them unfavorable from an economic point of view (Kim and Rajapakse, 2005).

## Chitin, Chitosan, and Chitooligosaccharides: General Characterization

**Figure 1:**
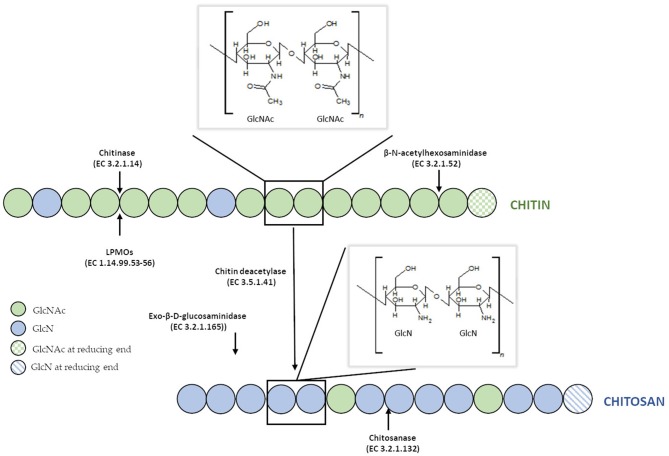
Structure of chitin and chitosan and their enzymatic modifications.

Chitin is defined as β-(1–4) linked linear cationic heteropolymer consisting of 2-acetamide-2-deoxy-D-glucopyranose (N–acetyl–D–glucosamine, GlcNAc) and randomly distributed units of 2-amino-2-deoxy-D-glucopyranose (D–glucosamine, GlcN) (Figure 1). The degree of chitin acetylation (DA) exceeds 90%, and its molecular weight (MW) can be as high as 1 × 10^6^-2.5 × 10^6^ Da, which corresponds to a degree of polymerization (DP) of ca. 5,000–10,000 (Mourya and Inamdar, 2008). The presence of acetamido groups enables the formation of numerous inter- and intramolecular hydrogen bonds between linear chitin chains. Moreover, these groups are situated near the hydroxyl groups in the *trans* position, which results in a high degree of crystallinity and lack of solubility in water and organic solvents. Research studies on chitin revealed that this biopolymer displays many unique properties. For example, it inhibits the growth of bacteria, fungi and viruses, has a tremendous chelating ability, exhibits high affinity for proteins, it, therefore, makes it valuable for immobilization of enzymes (Synowiecki and Al-Khateeb, 2003; Younes and Rinaudo, 2015). Due to its biodegradability, biocompatibility, non-toxicity, physiological inertness and gel-forming properties, chitin has found countless applications in different industries, e.g., food, cosmetic, pharmaceutical, manufacturing of synthetic materials, agriculture, and even electronics for the production of biosensors (Rinaudo, 2006).

Unfortunately, due to the lack of solubility and its highly ordered crystalline structure resistant to physical and chemical agents, the use of chitin is limited in many cases. Therefore, the N-deacetylated derivative of chitin—chitosan, which is soluble in aqueous solutions of both organic and inorganic acids was found to have a practical advantage. This cationic polymer also consists of β-(1–4) linked N–acetyl–D–glucosamine and prevailing D–glucosamine residues. Chitosan does not refer to a single unique substance, but rather to many copolymers with a different ratio of GlcN to GlcN and GlcNAc residues). The degree of deacetylation (DD) of commercial preparations of chitosan are within 70–95%, and their MW ranges from 10^4^ to 10^6^ g/mol (Moura et al., 2011). Chitosans exists as a heterogenic group of oligomers and polymers which differ in their degree of polymerization (DP), a fraction of acetylation (FA), and their pattern of acetylation (PA) (Kohlhoff et al., 2017). Compared to chitin, this N–deacetylated derivative is much less common in nature and is manufactured industrially by hydrolyzing amino acetyl groups of chitin. The polymer is also naturally present in the cell walls of filamentous fungi primarily classified to the *Zygomycete*s class (Synowiecki and Al-Khateeb, 1997; Chatterjee et al., 2005). Despite significant similarities in the molecular structures of chitin and chitosan, the physicochemical characteristics of both biopolymers and the reactions they undergo are often surprisingly distinct. Both polymers possess reactive hydroxyl and amino groups (in different molecular ratios), but lower crystallinity of chitosan makes it more accessible for reagents (Mourya and Inamdar, 2008). Probably, the most crucial difference between chitin and chitosan in terms of their applications is in their DD and solubility. Chitosan is soluble in most aqueous acid solutions such as acetic, formic, lactic, citric acids below its pKa (pH around 6.5), and some others solvents such as dimethylsulfoxide, p-toluene sulfonic acid (Roy et al., 2017). The chemical modification of molecular structure of chitosan can significantly improve the solubility of the polymer in different solvents. Yang et al. (2015) synthesized chitosan-graft-polycaprolactone which was soluble in dimethylformamide (DMF), dimethyl sulfoxide (DMSO), ethanol and toluene. O-alkylated chitosan synthesized in an ionic liquid solvent was soluble chloroform, ethanol, water and acetic acid (Chen H. et al., 2012). Monomethyl-modified chitosan obtained by modification of chitosan with monomethyl fumaric acid in an ionic liquid solution, showed excellent solubility in water, which significantly increased the spectrum of its applications (Wang et al., 2015).

The biological applications of chitin and chitosan are highly limited due to their high molecular weight, poor solubility and high viscosity of chitosan solutions. Chitooligosaccharides (COS), which are the products of chitin and chitosan degradation, are seen as an excellent alternative. COS also consists of β-(1–4) linked N–acetyl–D–glucosamine and prevailing D–glucosamine residues. The DP of COS varies from 2 to 20 units in a segment which gives an average MW lower than 3,900 Da. Each oligosaccharide differs in the FA and in the sequence of GlcN and GlcNAc residues. Heterochitooligosaccharides are composed of GlcN and GlcNAc comprising both units, while homochitooligosaccharides are exclusively composed of GlcN or GlcNAc. Heterooligosaccharides differ in the DP, DD, and position of N-acetyl residues in the oligomer chain (Liaqat and Eltem, 2018). The water solubility of COS is associated with their shorter chain lengths and free amino groups in D-glucosamine units (Bahrke, 2008). The solubility of COS depends on the degree of polymerization. For example, oligomers with DP 2–4 are soluble in methanol but oligomers with DP more than 5 are less soluble (Mourya et al., 2011). However, it is generally assumed that COS are insoluble in ethanol, propanol, butanol, acetone, ethyl acetate, partially soluble in methanol, and dimethyl sulfoxide but fully soluble in water (Liaqat and Eltem, 2018). Because of the higher solubility than chitin and chitosan in generally available solvents COS have gained an increasing interest in many laboratories and industries. The biological activity of oligosaccharides depends on their structure and is mainly dependent on their DP, MW, DA, FA, and PA (Li et al., 2016).

## Chitin and Chitosan Production Methods

### Chitin Production Methods

The primary source of raw materials to produce chitin and its N-deacetylated derivatives are wastes of the fishing industry. Exoskeletons of marine organisms, including shrimp, crab, crayfish, krill, squid, are widely used for this purpose (Abdou et al., 2008). Nowadays, chitin and chitosans are obtained by two types of extraction methods: chemical and biotechnological. Chemical processes which involve the use of strong acids and bases are currently the most widely used methods in both laboratory and industrial-scale production.

The process of chitin extraction and its transformation into chitosan includes three major steps: demineralization, deproteinization, and deacetylation. Additionally, decolorization process using various organic and inorganic solvents such as glacial acetone (Soon et al., 2018), sodium hypochlorite (Srinivasan et al., 2018) can be employed to eliminate pigments. Demineralization step is performed to remove the calcium carbonate and calcium chloride, which are the main inorganic constituents of the exoskeletons of crustaceans. For this, inorganic acids such as HCl, HNO_3_, and H_2_SO_4_ (Kumar Gadgey and Bahekar, 2017), and strong organic acids HCOOH and CH_3_COOH (Regis et al., 2015) are used. The most common acid used in the production of chitin is hydrochloric acid, due to its high efficiency in the removal of the minerals. The next major step in chitin extraction is deproteinization of raw materials. This step is performed using alkali solution to remove proteins. A wide range of chemical reagents have been tested for protein removal including NaOH, Na_2_CO_3_, NaHCO_3_, KOH, K_2_CO_3_, Ca(OH)_2_, Na_2_SO_4_, NaHSO_4_, CaHSO_4_, Na_3_PO_4_, and Na_2_S (Younes and Rinaudo, 2015). However, the most commonly used is NaOH solution. The chemical extraction of chitin involves large amounts of hazardous alkaline and acid wastes which are dangerous for the environment. Biological methods offer an alternative way to extract chitin and chitosan. Many research results are indicating that the chemical deproteinization of chitin can be replaced by enzymatic methods. Hamdi et al. (2017) extracted chitin from blue crab *Portunus segnis* and shrimp *Penaeus kerathurus* using chemical demineralization and enzymatic deproteinization approaches. The use of *P. segnis* crude extract with proteolytic activity resulted in deproteinization degree (DP) of 85 and 91% for blue crab and shrimp, respectively. Castro et al. (2018) extracted chitin from *Allopetrolisthes punctatus* crab biomass using biotechnological method. Demineralization and deproteinization of crab biomass were carried out using lactic acid fermentation, by *Lactobacillus plantarum* sp. 47. Extracted and purified chitin, after 60 h fermentation, showed 99.6 and 95.3% demineralization and deproteinization, respectively, using low concentrations of acids and bases. Dun et al. (2019) presented a new strategy for chitin extraction by simultaneous enzymatic hydrolysis and fermentation. *Bacillus coagulans* LA204 and proteinase K were used to remove minerals and proteins from crayfish shell waste powder. After 48 h of fermentation, the deproteinization efficiency, demineralization efficiency, and chitin recovery reached 93, 91, and 94%, respectively. Despite the many disadvantages of chemical methods, they are still prevalent approach for chitin processing in the industrial most likely due to short extraction time. Table 1 shows a comparison of chemical and biotechnological methods of chitin production.

**Table 1:** Chitin production methods.

**Method**	**Chitin production**							
	**Source**	**Demineralization**	**DM [%]**	**Deproteinization**	**DP [%]**	**References**	**Advantages**	**Disadvantages**
Chemical process	Resting eggs *Zophobas morio* larvae Shrimp shells	1:50 (w/v); 1 M HCl 16 h, 65–75°C 1:30 (w/v); 1 M HCl75 min, RT, 150 RPM	NI – –	1:5 (v/v); 1 M NaOH 20 h, 65°C 1:20 (w/v); 1 M HCl 30 min, 100 RPM, 35°C 1:20 (w/v); 0.5–2 M NaOH 20 h, 80°C, 100 RPM 1:30 (w/v); 3M NaOH75 min, RT, 150 RPM	NI – –	Kaya et al., 2013 Soon et al., 2018 Srinivasan et al., 2018	Short processing time; Used at the industrial scale; Complete removal of organic salts;	Environmentally unfriendly; Solubilized minerals and proteins cannot be used as human and animal nutrients; Uncontrolled hydrolysis of the product;
Enzymatic deproteinization and chemical demineralization	Blue crab	1:10 (w/v); 0.55 M HCl 30 min, 4°C	NI	20 g Crab/Shrimp shell powder, crude protease extract from *P. segnis* pH 8.0, 50°C, 3 h	~85	Hamdi et al., 2017	Limited amount of hazardous waste for the environment; Complete removal of organic salts;	Solubilized minerals and proteins cannot be used as human and animal nutrients; Uncontrolled hydrolysis of the product; Relatively long processing time; Limited to laboratory-scale;
	Shrimp		NI		~91			
	Shellfish powder	1:2 (w/v); 1.5 N HCl 2 h, RT	99	10 g powder; crude protease extracted from *Erwinia chrysanthemi* 37°C, 16 h	~95	Sami, 2010		
	Shrimp	1:10 (w/v) 1.5 M HCl 6 h, 25°C	100	1:20 crude protease extracted from *B. cereus* SV1 to pretreated shrimp wastes pH 8.0, 3 h, 40°C	~89	Manni et al., 2010		
Liquid fermentation and chemical demineralization	Shrimp shells	1:10(w/v); 0.5 M HCl 3 baths, 4°C, 30 RPM, 30 min	100	Two crude enzymes in separate reactions crude protease from *Bacillus mojavensis* A21–7.75 U/mg, 60°C, 6 h, pH 9.0 crude protease from *Scorpaena scrofa* 10 U/mg, 50°C, pH 9.0	96	Younes et al., 2016		
	Shrimp	1:50 (w/v); 1.25 M HCl 1 h, RT	NI	*Brevibacillus parabrevis* grown on medium containing 3%(w/v) shrimp waste, 37°C, 150 RPM, 5 days	73–96^*^	Doan et al., 2019b		
Liquid fermentation	Crab shells	*Lactobacillus plantarum* sp. 47 grown on medium containing 85% (w/v) raw material 32°C, 60 h		~95	~99	Castro et al., 2018	High quality of the final product; Environmentally safe; Removed minerals and proteins may be used as human and animal nutrients; Products with predicted physicochemical properties	Long processing time; Limited to laboratory-scale;
	Shrimp wastes	*Lactobacillus brevis* – solid state fermentation on minced shrimp wastes 30°, 192 h		96	67.3	Aranday-García et al., 2017		
	Shrimp waste	*Pseudomonas aeruginosa* grown on medium containing 5% (w/v) shrimp 37°C, 100 RPM, 144 h		92	82	Sedaghat et al., 2017		
	Shrimp head	*Streptococcus thermophilus* grown on medium containing 10% (w/v) shrimp head 42°C, 64 h, pH 5.00		~94	92	Mao et al., 2013		
Simultaneous enzymatic hydrolysis and fermentation	Shell waste	*Becillus coagulans* LA204 grown on medium containing 5%(w/v) crayfish shell powder (CSP) and 1,000 U proteinase K kg^−1^ (CSP) 50°C, 48 h		93	91	Dun et al., 2019		

* *Depending on the shrimp source*.

### Chitosan Production Methods

Currently, there are two well-known methods of chitosan preparation. The first approach is to extract chitosan directly from cell walls of molds. The second approach utilizes thermo-chemical or enzymatic methods of chitin deacetylation to remove the N-acetyl groups from chitin. The first microbiological method of chitosan preparation was developed by White, Foulton and Farin in 1979 (Da Silva Amorim et al., 2001). Despite superior properties of microbiological chitosan, such as more efficient sorption of metals, compared to its counterparts obtained by the chemical N-deacetylation of chitin, this method did not find widespread application in industry. The main disadvantage of this approach is the insufficient amount of chitosan extracted from the cell walls of microorganisms. The chitosan content depends on the strain and ranges from 0.3 g (*Mucor rouxii* DSM 0201) (Davoust and Hansson, 1992) to 1.8 g (*Absidia coerulea* ATCC 14076) (Jaworska and Konieczna, 2001) per 1 dm^3^ of culture medium.

Nowadays, chitosan is manufactured industrially through thermo-chemical hydrolysis of chitin's amide bonds. Commercial preparations of chitosan are available in several forms such as solutions, flakes, fine powder, beads, and fibers (Hayes, 2012). Thermo-chemical methods of converting chitin to chitosan are used extensively on an industrial scale because of their low costs and suitability for mass production. In principle, chitin can be deacetylated using either acids or alkalis. Since glycosidic bonds are very susceptible to acid hydrolysis; the alkali-catalyzed deacetylation is used more frequently to avoid unwanted chain termination (Younes and Rinaudo, 2015). For this purpose, 50% NaOH solution is most often used at high temperature (Soon et al., 2018; Srinivasan et al., 2018). It had to be mentioned that the characteristics of extracted chitosan differ depending on the extraction method and the source from which chitin is isolated (Marei et al., 2016). Samar et al. (2013) improved conventional deacetylation method by adding of microwave irradiation. Obtained degree of deacetylation reached 95% with 90% chitosan yield. El Knidri et al. (2016) replaced the conventional method of chitin extraction and its transformation into chitosan by an alternative process intensified with microwave irradiation in all production steps: demineralization, deproteinization and deacetylation. Obtained results showed that chitosan, with a DD of 82.73%, was successfully prepared in 24 min *via* microwave irradiation method, while a much longer time of 6–7 h was needed for preparing chitosan with the same degree of deacetylation (DD = 81.5%), using a conventional heating method. Despite a relatively high efficiency of thermo-chemical methods, these processes have many disadvantages, such as high-energy consumption and a large amount of waste alkaline solution resulting in environmental pollution. Many factors impact the basic properties of obtained chitosans, such as the process duration (Tsaih and Chen, 2003), the temperature and number of repetitions of alkaline steps (Tolaimate et al., 2000), and the concentration and type of alkali reagent (Younes et al., 2014). These methods are very difficult to control, resulting in a mixture of heterogeneous species with different physicochemical properties that are difficult to fractionate into desired products with known characteristics. Additional factors such as the presence and type of reducing agent, the gases constituting the reaction environment (nitrogen or air), particle size, and the source of raw material may influence the degree of deacetylation and the average molecular weight of the obtained products (Younes and Rinaudo, 2015). Younes et al. (2014) investigated that sodium borohydride (reducing agent), and nitrogen atmosphere do not have a significant effect on the DA of chitosan but was found to have a protective effect against chitosan degradation during deacetylation. Moreover, the acetyl groups of the resulting products are distributed irregularly with certain hot spots of acetylation. It has a significant impact on the solubility and properties of obtained chitosans (Aiba, 1991). The most significant disadvantage of these methods lies in uncontrolled hydrolysis of chitin, which occurs spontaneously, following the treatment with concentrated alkali and high temperature. A study carried out by Struszcyk (2000) demonstrated that the decrease in MW could reach even 86% in comparison to MW of the used substrate. It has been found that the use of oxygen scavengers and reducing agents, such as thiophenol and sodium borohydride (NaBH_4_), may limit polymer degradation (Younes and Rinaudo, 2015). However, the consequence is the increase in the process costs and the amount of hazardous chemical waste.

To overcome these problems, enzymatic methods can be used. Due to the eco-friendly nature of these methods, they attract significant interest. Moreover, the use of enzymes prevents irregular deacetylation and molecular weight reduction caused by acid and alkali treatment. Rass-Hansen et al. (2007) used chitin deacetylase isolated from *Colletotrichum lindemuthianum* for deacetylation of pre-treated chitin substrates extracted by liquid fermentation using microorganisms mixture consisting of *Lactobacillus salvarius, Enteroccus facium* and *Pedoicoccus acidilactici* strains. The main disadvantage of the enzymatic method is that the enzyme preparations are not able to efficiently deacetylate native chitin substrates. Pareek et al. (2013) proved that the efficiency of enzymatic deacetylation is strictly dependent on the method of pre-treatment of the substrate. Depending on the form of substrate preparation, they obtained a degree of deacetylation of the product in the range from 62 to 79%. Table 2 shows a comparison of chemical and biotechnological methods of chitosan production.

**Table 2:** Chitosan production methods.

**Method**	**Chitosan production**								
	**Source**	**Demineralization**	**Deproteinization**	**Deacetylation**	**DD [%]**	**Chitosan yield [%]**	**References**	**Advantages**	**Disadvantages**
Chemical process	Locus	1:15 (w/v); 1 M HCl	1:15 (w/v); 1 M NaOH 8 h, 100°C	50% NaOH 8 h, 100°C	98	–	Marei et al., 2016	Short processing time; Used at the industrial scale; Complete removal of organic salts;	Environmentally unfriendly; a large volume of concentrated alkali solution at high temperature Solubilized minerals and proteins cannot be used as human and animal nutrients; Uncontrolled hydrolysis of the product;
	Honey bee				96				
	Beetles				95				
	Shrimp				75				
	*Zophobas morio* larvae	1:20 (w/v); 1 M HCl 30 min, 100 RPM, 35°C	1:20 (w/v); 0.5–2 M NaOH 20 h, 80°C, 100 RPM	50% NaOH 30 h, 90°C	65–81	66	Soon et al., 2018		
	Shrimp shells	1:30 (w/v); 1 M HCl 75 min, RT, 150 RPM	1:30 (w/v); 3 M NaOH 75 min, RT, 150 RPM	1:50 (w/v); 50% NaOH 50 min, 90°C	–	35	Srinivasan et al., 2018		
Chemical process combined with microwave techniques	Cuttlefish pens	1:40 (w/v); 1 M HCl 3 h, RT	1:20 (w/v); 1 M NaOH 24 h, 70°C	1:15(w/v); 45% NaOH 15 min, 600 W	93	–	Al Sagheer et al., 2009	Limited amount of hazardous waste for the environment; Complete removal of organic salts;	Solubilized minerals and proteins cannot be used as human and animal nutrients; Uncontrolled hydrolysis of the product; Relatively long processing time; Limited to laboratory-scale;
	Shrimp waste	1:10 (w/v); 2% HCl 12 h, 30°C	1:10 (w/v); 4% NaOH 12 h, 90°C	1:10; 50% NaOH 1,400 W, 10 min, 60 mesh	~95	~90	Samar et al., 2013		
	Shrimp shells	1:10 (w/v); 3 M HCl 8 min, 500 W	1:10 (w/v); 10 NaOH 8 min, 160–350 W	1:20 (w/v); 50% NaOH 8 min, 350 W	~83		El Knidri et al., 2016		
Liquid fermentation and chemical deacetylation	Shrimp waste	*Pseudomonas* *aeruginosa* grown on medium containing 5% (w/v) shrimp 37°C, 100 RPM, 144 h		50% NaOH in an autoclave 50% NaOH, 100°C 50% NaOH microwaves	88 ~77 ~44	~88	Sedaghat et al., 2017	Limited amount of hazardous waste for the environment; Removed minerals and proteins may be used as human and animal nutrients;	Long processing time; Limited to laboratory-scale;
Liquid fermentation and enzymatic deacetylation	Minced prawn shell	*Lactobacillus salvarius, Enteroccus facium* and *Pedoicoccus acidilactici* 30°C, 250 RPM, 120 h		Pre-treated in different way chitin substrates were mixed with chitin deacetylase from *Colletotrichum lindemuthianum* 1:1 (v/v); 24 h, 50°C	–	–	Rass-Hansen et al., 2007	High quality of final product; Environmentally safe; Removed minerals and proteins may be used as human and animal nutrients;	Long processing time; Limited to laboratory-scale;
Chemical and enzymatic deacetylation	Commercial chitin	45% phosphoric acid, 40 min; Precipitation by addition of 6 M NaOH to pH 8. Cold solution of 20% NaOH and 0.2% SDS, 60 min, 4°C Overnight at −20°C and neutralized with 6 N HCl 20% HCl, 20 min, RT Precipitation in cold distilled water 100 ml of 60% calcium chloride in methanol solution Precipitation in 1% calcium citrate 25% formic acid, RT		Pretreated chitin deacetylated by chitin deacetylase from *Penicillium oxalicum* SAE_M_-51 24 h, 50°C	62 – 72 79	–	Pareek et al., 2013	Relatively short processing time; Highly deacetylated products; Limited amount of hazardous waste for the environment;	Limited to laboratory scale;

Despite numerous advantages of biotechnological methods, which undoubtedly include: the ability to control the process (no uncontrolled degradation of the polymer chain), desired physicochemical properties of the obtained products, no negative impact on the environment; conventional methods of chitin and chitosan production are still the most commonly used commercially because of their short extraction time and high efficiency of the process.

### Chitooligosaccharides Production Methods

As it was mentioned the biological activities of COS are significantly influenced by the DA, DP, MW, FA, and PA; therefore, it is crucial that the chitooligosaccharides production methods are reproducible and easy to control. At present COS can be obtained through physical, chemical, electrochemical and enzymatic degradation of chitin and chitosan. Currently, commercially available chitooligosaccharides are usually prepared by hydrolysis of chitin with concentrated acids and enzymatic hydrolysis of chitin and chitosan. The most commonly used chemical methods of chitooligosaccharides production include acid degradation and oxidative degradation of chitin and chitosan. Trombotto et al. (2008) described the two-step method of obtaining homogeneous series of chitin/chitosan oligomers varying from DA 0–90% with a narrow distribution of DPs within 2 and 12. The first step includes chemical depolymerization of fully deacetylated chitosan using 12 M hydrochloric acid. The obtained COS were partially N-acetylated in hydroalcoholic solution of acetic anhydride. It has been demonstrated that COS production can be carried out in nitrous acid environment (Tømmeraas et al., 2001). Another example of chemical depolymerization of chitinous substrates is the use hydrogen peroxide. Chitooligosaccharides with DP ranging from 2 to 9 were produced using H_2_O_2_ using phosphotungstic acid as a catalyst in homogenous phase (Xia et al., 2013). Due to the relatively high efficiency and simplicity of the process, chemical hydrolysis is the most frequently used traditional chitin and chitosan digestion method. However, chemical reactions are difficult to control and can lead to the production of various by-products which are problematic in the purification of the chitooligosaccharides. Besides, chemical degradation leads to a mixture of products of varying DP and DA.

Enzymatic methods, which are an excellent alternative to conventional processes, gained interest as precise tools for the production of COS with desired physicochemical and biological properties. Enzymatic production of COS can be carried out by using specific enzymes such as chitinases, chitosanases, and non-specific enzymes like carbohydrases and proteases (Kim and Je, 2010). Rafael Olicón-Hernández et al. (2017) described enzymatic hydrolysis of colloidal chitin using extracellular chitosanase from *Bacillus thuringensis*. Obtained mixtures of chitooligosaccharides obtained by *in vitro* depolymerization of colloidal chitin substrates consist of the chitobiose to the chitohexaose. Chitin from fungal waste mycelia pre-treated by alkali was degraded by recombinant chitinase from *Lactococcus lactis*. The main product in the obtained hydrolysate was N,N′-diacetylchitobiose. The yield of the product from waste mycelium was around 10% with estimated purity of around 70%. Addition on snailase into the reaction mixture significantly increased the yield COS to 24% with purity of 78% (Lv et al., 2016). Mallakuntla et al. (2017) described chitinase from *Enterobacter cloacae* which exhibit transglycosylation activity. The profile of hydrolytic products among which predominant was chitobiose, indicated that the chitinase was an endo-acting enzyme. Transglycosylation reaction catalyzed by chitinase resulted in the formation of longer chitooligosaccharides by joining trimeric to hexameric COS for a prolonged duration. As already mentioned, COS can also be produced by hydrolysis of chitin and chitosan using non-specific enzymes. Xie et al. (2011) used complex enzymes compound of commercial cellulose, pectinase and α-amylase to degrade the large molecular chain of chitosan rapidly under slightly acidic conditions. Results indicated that complex enzymes could result in low molecular weight chitosan ranged from 1,000 to 4,000 after enzymatic degradation for 2 h without changing chitosan glycosidic ring structure and DD.

There have been many reports indicating that ultrasonic microwave and gamma rays could be used to prepare COS with little contamination but, unfortunately with relatively low proactivity. Popa-Nita et al. (2009) evidenced the existence of two mechanisms involved in the ultrasonically induced chitosan depolymerization. The effect of the first is the rapid disruption of polymer chains while reducing polydispersity. The second mechanism is different and results in formation of short polymer chains and oligomers with an increase in polydispersity. Using different conditions of ultrasonic depolymerization COS ranging from DP 2–11 with maximum concentration of DP 3 were obtained. Baxter et al. (2005) suggested that ultrasonic treatment of chitosan in the medium to low power range can replace chemical and enzymatic methods that are currently used to depolymerize chitosan. The results of their research indicate that in the presence of acetic acid ultrasonication can be utilized to reduce molecular weight of chitosan while maintaining the degree of acetylation. Gamma irradiation was also successfully used to depolymerize chitosan. Choi et al. (2002) applied Co-60 gamma irradiation to depolymerize chitosan in acetic acid solution. The rapid decrease in viscosity of the solution was observed with the production of COS wit DP ranging from 2 to 4. In recent years, hydrodynamic cavitation has gained considerable interest in the context of polymer degradation. Depolymerization takes place as the liquid is passed through a constriction, which leads to higher bubble densities and subsequent increase in local pressure. Wu et al. (2014) degraded chitosan by swirling cavitation, which is a variation of hydrodynamic cavitation. The intrinsic viscosity reduction rate of chitosan was 83.65% with no change in the DD of the products. So far, physical methods of COS production have not been investigated too frequently and their large-scale efficiencies have not been established. An interesting alternative to the COS production methods described so far is electrochemical method, which is easy to control and contamination free. Unfortunately, there are some significant problems such as the short electrode life and easy failure. Gu et al. (2013) used Ti/Sb–SnO_2_ electrode as anode, stainless steel as cathode for degrading chitosan. The molar mass of chitosan dramatically decreased with reaction time and the chemical structure of degraded chitosan was not obviously modified. The above-described methods for the production of COS have their advantages and disadvantages. The Table 3 lists the most important of them.

**Table 3:** Advantages and disadvantages of chitooligosaccharides production methods.

**COS production method**	**Advantages**	**Disadvantages**
Chemical depolymerization	High efficiency simple to handle	Harmful to the environment difficult to control lead to the production of various by-products mixtures of products of varying DP and DA
Physical depolymerization	Easy purification and little contamination lack of waste harmful to the environment	Low productivity energy-consuming
Enzymatic depolymerization	Easy to control easy accessibility products with desired properties no additional products modification	High costs of enzymes preparations
Electrochemical depolymerization	Easy to operate contamination-free	Short electrode life easy to fail

## Enzymatic Modification of Chitin and Chitosan

**Figure 2:**
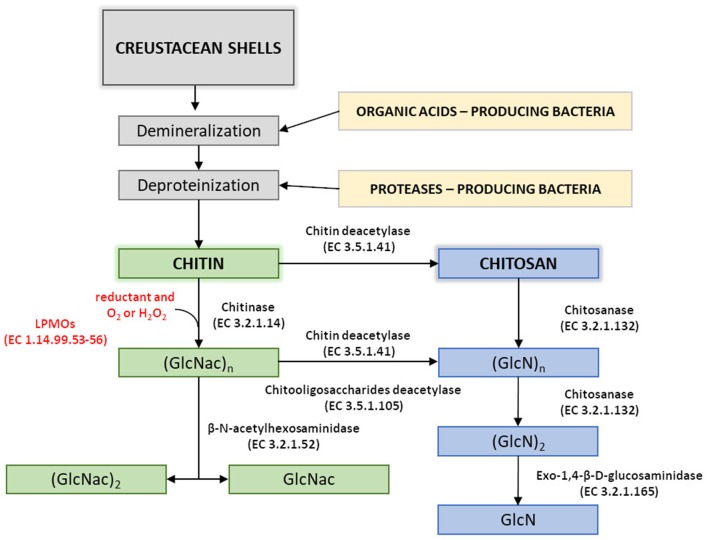
Enzymatic pathways for chitin and chitosan modification. Figure adapted and expanded from Jung and Park (2014). Distributed under the terms of the Creative Commons Attribution License.

Disadvantages of currently used thermo-chemical methods of chitin and chitosan industrial-scale manufacturing and increasing demand for a range of their derivatives with fully defined architecture have drawn attention to the enzymatic methods of polymer modification. Enzymatic pathways for chitin and chitosan conversion are shown in Figure 2 (Jung and Park, 2014).

Efficient conversion of chitin into specific chitosan can be catalyzed by chitin deacetylases (EC 3.5.1.41, ChDa). According to the classification of enzymes, a group of enzymes that catalyze the COS deacetylation reaction should also be distinguished. Chitin deacetylases and chitooligosaccharides deacetylases [EC 3.5.1.105 (CODa)]. ChDas and CODa are a group of enzymes catalyzing the hydrolysis of acetamido groups of N–acetyl–D–glucosamine residues in chitin, chitosan and chitooligosaccharides, respectively (Li et al., 2007; Pacheco et al., 2013). Both of these groups of enzymes are classified in the carbohydrate esterase family 4 (CE4) in the CAZY database (Lombard et al., 2014). These enzymes share a conserved region known as the NodB homology domain due to its similarity to the NodB oligosaccharide deacetylase, one of the first CE4 enzymes to be characterized (John et al., 1993). The activity of these enzymes has been discovered in several fungi, marine bacteria, and insects (Tsigos et al., 2000; Hirano et al., 2015). Chitin and chitooligosaccharides deacetylases have a wide range of molecular masses, ranging from 12 to 150 kDa. The optimum temperature for the activity of most ChDa varies from 30 to 60°C (Grifoll-Romero et al., 2018). Fungal ChDa can exist as intracellular (e.g., from *Mucor rouxii, Absidia orhidis*) or extracellular enzymes (e.g., produced by *Mucor circinelloides, Colletotrichum lindemuthianum, Aspergillus nidulans*) (Table 4) (Jaworska, 2012; Kaczmarek et al., 2016). Thanks to the catalytic capabilities of these enzymes, and significant thermal stabilities ChDa are more often seen as suitable tools for biotechnological chitosan production. Currently, the challenge is to develop enzymatic methods that enable the conversion of the native form of chitin to its deacetylated derivatives. Martinou et al. (1997) indicated that two chitin deacetylases isolated from *Absidia coerulea* and *Mucor rouxii* are not able to efficiently modify native chitin. They suggest that the pre-treatment of crystalline chitin is, therefore, a necessary step before addition of an enzyme in order to improve the accessibility of the acetyl groups and to enhance the yield and the rate of the deacetylation reaction. A contrasting study conducted by Win and Stevens (2001) showed that the pre-treatment of chitin using various physical and chemical conditions did not result in a more efficient enzymatic deacetylation catalyzed by ChDa from *Absidia coerulea*. However, decrystallised chitin superfine (SF) obtained by dissolution of native chitin in specific solvents followed by fast precipitation, pre-treated with 18% formic acid, appeared to be a suitable substrate for fungal deacetylase. In this way, chitin (10% DD) was deacetylated by the enzyme into chitosan with DD of 90%. Kim et al. (2008) in his research tested a variety of substrates such as crystalline-β-chitin, chitin and chitosans with different DD, water-soluble chitin (DD 50%) (WSCT-50), glycol chitin and other chitin derivatives. Obtained results indicated that chitin deacetylases isolated from *Mortierella* sp. are only capable of the efficient deacetylation of WSCT-50, glycol chitin, and crab chitosan (DD 71) with a relative activity 100, 35, and 49%, respectively. However, Tuveng et al. (2017) described extracellular ChDa from marine *Arthrobacter* species which showed activity against insoluble β-chitin. Unfortunately, no efficient enzymatic method of deacetylation of crystalline, native chitin has been developed so far.

**Table 4:** The biochemical properties of known fungal chitin deacetylases.

**Organism**	**pH optimum**	**Temp. optimum (^**°**^C)**	**Molecular mass (kDa)**	**Activation**	**Inhibition**	**Substrate specificity**	**References**
*Pestalotiopsis* sp.	8.0	55	ND	–	Fe^2+^, Mn^2+^	Active against: soluble chitosan, colloidal chitin the activity increases with the increase of the DA of the substrate inactive against: insoluble α-chitin and β-chitin	Cord-Landwehr et al., 2016
*Colletotrichum lindemuthianum*	11.5–12.0	60	33	Co^2+^, Zn^2+^ (1 mM)	Co^2+^ (10 mM), Ni^2+^, Fe^2+^, Cu^2+^, Mn^2+^	active against: glycol chitin, partially N-deacetylated water soluble chitin, chitin oligomers inactive against: N-acetylglucosamine.	Tokuyasu et al., 2009
*Rhizopus circinans*	5.5–6.0	37	75	Mn^2+^, Mg^2+^	Cu^2+^	Active against: glycol chitin, partially deacetylated chitin, native chitin, low activity against chitohexoses, colloidal chitin	Gauthier et al., 2008
*Aspergillus nidulans CECT 2544*	7.0	50	27	–	Cd^2+^, Co^2+^ Ag^2+^, Ca^2+^, Sn^2+^, Pb^2+^ Zn^2+^, Mg^2+^, Mn^2+^ (40 mM)	Active against: glycol chitin, acetylated oligomers, chitin, colloidal chitin, α-1 → 3, 1 → 6-N-acetylgalactosamine-galactan	Alfonso et al., 1995
*Saccharomyces cerevisiae*	8.0	50	43	Co^2+^	Mg^2+^, Ca^2+^ Zn^2+^, Cu^2+^	Active against: hexa-N-acetylchitohexaose	Martinou et al., 2002
*Mucor rouxii* ATCC 24905	5.8	50	ND	Zn^2+^ Ca^2+^, Co^3+^	Mn^2+^, Fe^2+^ Fe^3+^	Active against: colloidal chitin, carboxymethylcellulose, crystalline chitin and dissolved chitosan with DA 6%.	Kołodziejska et al., 1999
*Scopulariopsis brevicaulis*	7.5	55	55	ND	ND	Active against: crystalline chitin, water-soluble chitosan (54% DD), N-acetyl-chitooligosaccharides with DP of 2–6, but not for GlcNAc	Cai et al., 2006

**Figure 3:**
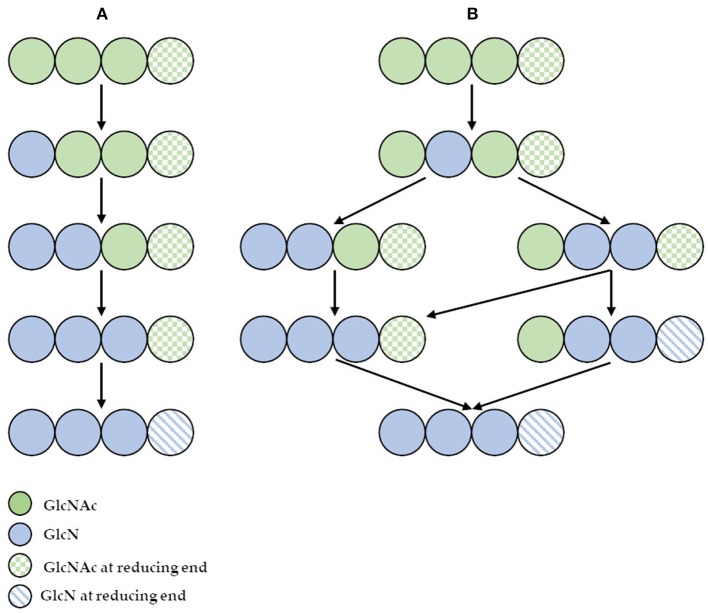
**(A)** The deacetylation of (GlcNAc)_4_ catalyzed by exo-type chitin deacetylase from *Mucor rouxii*—multiple attack mechanism; **(B)** the deacetylation of (GlcNAc)_4_ catalyzed by endo-type chitin deacetylase from *Colletotrichum lindemuthianum*—multiple chain mechanism. Figure adapted from Zhao et al. (2010).

However, the use of these enzymes can significantly reduce the dependence on conventional chitosan's production methods. It has been demonstrated that ChDa and CODa isolated from different sources exhibit different catalytic mechanisms, indicating that a variety of well-defined chitooligosaccharides can be produced during a single enzymatic reaction. The mechanism of action of enzymes that modify monomers within the polymer chain is commonly classified as multiple-attack, multiple chains, or single-chain mechanism (Figure 3) (Grifoll-Romero et al., 2018). For example, exo-type ChDa from *M. rouxii*, hydrolyses the acetyl groups of chitinous polymers such as glycol chitin, colloidal chitin, chitosan, chitin, and chitooligosaccharides (DP 1–7) from non-reducing end according to the progressive multiple-attack mechanism. The binding of the enzyme to the polysaccharide chain is followed by a number of sequential deacetylations, after which the enzyme binds to another region of the polymeric chain. This mechanism generates a block-copolymer structure with GlcN units within the GlcNAc chain (Araki and Ito, 1975; Martinou et al., 1998). Martinou et al. (1998) indicated that the length of the COS has a significant impact on enzyme activity. It has been shown that *M. rouxii* chitin deacetylase cannot effectively deacetylate COS with DP < 3. On the other hand (GlcNAc)_4_ and (GlcNAc)_5_ were fully deacetylated by the enzyme, while in the case of (GlcNAc)_3_, (GlcNAc)_6_, and (GlcNAc)_7_ the reducing-end residues were always intact. Fungal ChDa from *Colletotrichum lindemuthianum* was another enzyme with a thoroughly investigated mode of action. Unlike the ChDa from *M. rouxii* the *C. lindemuthianum* protein is an endo-type extracellular enzyme catalyzing the hydrolysis of acetamido groups according to a multiple-chain mechanism. The enzyme forms an active complex with the polymer chain and catalyzes the hydrolysis of only one acetyl group. After hydrolysis enzyme dissociates from complex and the cycle restarts. This mode of action generates binary heteropolysaccharides with a random distribution of deacetylated units (Blair et al., 2006). Tokuyasu et al. (1997) indicated that (GlcNAc)_3_ and (GlcNAc)_4_ were fully deacetylated, whereas the reducing-end residues (GlcNAc)_2_ could not be deacetylated. Recombinant chitin deacetylase from *Pestalotiopsis* sp. deacetylates all residues of the substrates (DP2-DP6) leaving unmodified GlcNAc residue at the reducing end and the last two GlcNAc units from the non-reducing end. Obtained results indicated that enzyme catalyzed the deacetylation reaction through a multiple-chain mechanism (Cord-Landwehr et al., 2016). Another example of an enzyme that works according to this mechanism is CE4 deacetylase isolated from a marine *Arthrobacter species* which was active against chitosan, acetylxylan, insoluble chitin and COS with DP ranging from 2 to 6 (Tuveng et al., 2017). In the single chain mechanism, the processive enzyme catalyzed a number of deacetylation reactions on a single substrate molecule leading to sequential deacetylation. Enzymes of bacterial origin, which are capable of deacetylation of chitooligosaccharides show this mechanism of action (Li et al., 2007). Chitooligosaccharides deacetylase described by Li et al. (2007) produced COS lacking only one acetyl group compared to the substrates. ChDa and CODa with diverse activities against chitooligosaccharides are owerful tools for the production of COS with desired properties. Table 5 presents examples of ChDa and CODa with well-characterized activities against COS with different DP.

**Table 5:** Examples of ChDa and CODa with characterized activity against COS with different DP.

**Source**	**ID Uniprot or GenBank**	**Substrates**	**Products PA**	**References**
*Aspergillus nidulans*	Q5AAA0	DP 2–5 activity increased with increased DP	DP2 GlcN-GlcNAc DP3-6 GlcN_n_	Liu et al., 2017
*Podospora anserine*	XP_001912680.1	Polymeric chitosans with different DAs	DP_n_ GlcN	Hoßbach et al., 2018
*Puccinia graminis*	XP_003323413.1	DP 4–6 activity increased with increased DP	GlcNAc-GlcNAc-GlcN_n−2_	Naqvi et al., 2016
*Pastolotiopsis* sp.	APH81274.1	DP 4–6	GlcNAc-GlcNAc-GlcN_n−3_-GlcNAc	Cord-Landwehr et al., 2016
*Pachonia chlamydosporia*		DP 4–6 non-active against DP <4	GlcNAc-GlcN-GlcN-GlcNAc_n−3_	Aranda-Martinez et al., 2018
*Vibrio cholerae*	Q9KSH6	DP 2–6 activity increased with decreased DP	GlcNAc-GlcN-GlcNAc_n−2_	Li et al., 2007
*Shewanella baltica*	ABN60929.1	DP 2–4; increase in activity DP2>DP4>DP3	DP2 GlcNAc-GlcN DP 3–4 GlcNAc-GlcN-GlcNAc_n−2_	Hirano et al., 2017
*Saccharomyces cerevisiae*		DP 4	GlcN-GlcNAc- GlcNAc- GlcNAc GlcNAc- GlcN- GlcNAc- GlcNAc GlcNAc- GlcNAc- GlcN- GlcNAc GlcN- GlcN- GlcNAc- GlcNAc GlcN- GlcNAc- GlcN- GlcNAc GlcNAc- GlcN- GlcN- GlcNAc GlcN- GlcN- GlcN- GlcNAc	Zhu et al., 2019

Presently, COS are manufactured industrially by chemical or physical depolymerization of the respective polymers (Aam et al., 2010; Mourya et al., 2011). Unfortunately, their production involves harsh thermo-chemical treatment, resulting in a high cost of processes and in large amounts of generated chemical wastes which are environmentally unfriendly. Moreover, the production is challenging to control and leads to broad heterogeneous mixtures. In this context, it should be mentioned that the biological and physicochemical properties of COS are also strongly dependent on the DA, DP, and MW. Jeon and Kim (2000) found that antibacterial and antifungal activity of COS grew with an increase of DP. Additionally, the inhibitory effect was much stronger for COS with lower DA than for those with a high degree of acetylation. It has been reported that COS with higher DA exhibited the highest ACE (angiotensin-converting enzyme) inhibitory effect, which prevents increases in blood pressure (Park et al., 2003). Huang et al. (2006) established that the molecular weight of COS also played a significant role in their antitumor activity. Thus, the attention is increasingly being focused on enzymatic methods, especially those using specific enzymes, such as chitosanases and chitinases (Abdel-Aziz et al., 2014). Generally, chitinases are defined as a group of enzymes that catalyze the cleavage of chitin. The enzyme nomenclature committee has defined **chitinases** (EC 3.2.1.14) as the enzymes capable of performing endohydrolysis of β-1,4-linkages in chitin. Many authors distinguish another subclass of chitinases, endochitinases—**β-N-acetylhexosaminidases** (EC 3.2.1.52), that catalyze the hydrolysis of terminal non-reducing N-acetyl-D-hexosamine residues in chitin (Duo-Chuan, 2006). Chitinases, which represent a class of glycosidic hydrolases, were found in many organisms, including viruses, bacteria, fungi, insects, higher plants, and mammals, in which they fulfill different functions (Karthik et al., 2014). Fungal chitinases have a wide range of molecular masses, ranging from 30 to 108 kDa. The optimum temperature for the activity of most fungal chitinases varies from 20 to 40°C. It has been reported that enzymes isolated from two thermophilic fungi *Thermomyces lanuginosus* (Zhang M. et al., 2015) and *Talaromyces emersonii* (McCormack et al., 1991) exhibit higher optimum temperature and thermostability. The recombinant Chit1 from *Thermomyces lanuginosus* exhibited optimum activity at 50°C and retained 56% of its activity at 60°C after 30 min, while Chit2 was optimally active at 40°C and retained 71% of its activity at 50°C after 60 min (Zhang J. et al., 2015). Table 6 shows examples of chitinases of microbial origin. Based on amino acid sequence similarity of catalytic domains, chitinases have been classified into glycosyl hydrolase families GH-18 and GH-19, which do not share a typical structure. GH-18 chitinases are characterized by a (β/α)_8_ barrel fold, while GH-19 chitinases are characterized by a high content of α-helices (Stoykov et al., 2015). These two families exhibit different mechanisms of catalysis. It has been shown that chitinases from the GH-18 family use retaining mechanism yield β-anomer hydrolysis products, whereas family GH-19 result in the α-anomer (an inverting mechanism) (Brameld and Goddard, 2002). Meanwhile, exochitinases, i.e., β-N-acetylhexosaminidases, have been assigned to the GH-20 family (Karthik et al., 2014). The GH-18 family chitinases are found in bacteria, fungi, yeast, viruses, plants and animals, while members of the GH-19 family have mostly been identified in plants. The GH-20 family includes bacterial and human chitinases (Duo-Chuan, 2006; Karthik et al., 2014). It has been proven that some organisms can produce more than one kind of chitinases. For example, the mycoparasite *Stachybotrys elegans* produces two exochitinases (β-N-acetylhexosaminidases) and one endochitinase (Taylor et al., 2003). Chitinases have the unique ability to hydrolyse GlcNAc-GlcNAc bonds makes these enzymes capable of hydrolysing chitin and to some extent, partially acetylated chitosan as well.

**Table 6:** The biochemical properties of chitinases.

**Organism**	**pH optimum**	**Temp. optimum (^**°**^C)**	**Molecular mass (kDa)**	**Activation**	**Inhibition**	**Substrate specificity**	**References**
*Paenibacillus pasadenensis* CS0611	5.0	50	69	–	Mn^2+^, Mg^2+^ and Co^2+^	Active against: colloidal chitin, chitin powder, crab shell powder Inactive against: chitosan, carboxymethyl cellulose and cellulose	Guo et al., 2017
*Streptomyces albolongus* ATCC 27414	5.5	55	47	Mn^2+^, Ba^2+^, Na^+^	Fe^3+^, Cu^2+^, Na_2_EDTA and SDS	Active against: colloidal chitin, chitin powder, chitosan, colloidal chitin	Gao et al., 2018
*Humicola grisea*	3.0	70	50	Mn^2+^, Co^2+^, ${\text{NH}}_{4}^{+}$ and Mg^2+^	Hg^2+^, Ca^2+^, Cu^2+^, K^+^ and EDTA	Active against: (GlcNAc)_2_, (GlcNAc)_3_ And colloidal chitin	Kumar et al., 2018
*Thermobifida fusca* reveals Tfu_0580	6.0	30	ND	Ca^2+^ and Mn^2+^	–	active against: (*p*-NP-(GlcNAc)_3_), (*p*-NP-(GlcNAc)_2_)	Yan and Fong, 2018
*Aspergillus terreus*	5.6	50	60	Ca^2+^, Mn^2+^ and Na^2+^	Cd^2+^, Zn^2+^, pb^2+^ and Hg^2+^	ND	Farag et al., 2016
*Penicillium* sp. LYG 0704	5.0	50	41	Mg^2+^ and Mo^2+^	Fe^2+^ and Hg^2+^	ND	Lee et al., 2009

Song et al. (2018) investigated the degradation patterns of chitin oligosaccharides using chitinase purified from pear pollen *Pyrus bretschneiderilia*. During 24-h reaction the enzyme acted as an endo-type chitinase and effectively degraded (GlcNAc)_5_ and (GlcNAc)_6_ to (GlcNAc)_2_, (GlcNAc)_3_, (GlcNAc)_4_; and to (GlcNAc)_2_, (GlcNAc)_3_, (GlcNAc)_4_, (GlcNAc)_5_, respectively. However, no degradation occurred for (GlcNAc)_2_ and (GlcNAc)_3_. The highest enzymatic activity was observed at 37°C, at pH 3 after a 3-h incubation. Chitinase from thermophilic *Humicola grisea* was studied by Kumar et al. (2017). Thin-layer chromatography (TLC) revealed that the enzyme could effectively produce COS using colloidal chitin as a substrate. After 30 min of incubation at 60°C a substantial increase in (GlcNAc), (GlcNAc)_2_, and (GlcNAc)_3_ was noticed. Prolonged incubation (up to 240–300 min) resulted in a further increase in (GlcNAc) concentration. Another study on chitinase was conducted by Moon et al. (2017). They used partially purified chitinase from *Serratia marcescens* to investigate the patterns of degradation of (GlcNAc)_2−4_. With (GlcNAc)_4_ as a substrate, the highest concentration of (GlcNAc)_1−3_ was obtained at 50°C, whereas at 70°C only (GlcNAc)_2_ was detected. Moreover, TLC analysis revealed that the most substantial amount of (GlcNAc)_1−3_ was produced at pH 5.0–6.0. The obtained results indicated that the reaction conditions may have a significant impact on the mode of the action of a biocatalyst.

**Chitosanases** (EC 3.2.1.132) constitute a family of enzymes capable of performing endohydrolysis of a β-1,4-glycosydic bond between GlcN residues in partially acetylated chitosan, from the reducing end. There is also another class of enzymes, **exo-β-D-glucosaminidase** (EC 3.2.1.165) that attack chitosan from its non-reducing end (Thadathil and Velappan, 2014). The activity of chitosanases has been observed in many different microorganisms, including bacteria, cyanobacteria, fungi, and plants (Thadathil and Velappan, 2014) in which they occur as intra- or extracellular enzymes. Most bacteria and fungi secrete chitosanases extracellularly except for fungi belonging to the class *Zygomycetes* [e.g., *Absidia orchidis* (Jaworska, 2012), *Mucor rouxii* (Alfonso et al., 1992), *M. circinelloides* (Struszczyk et al., 2009; Struszczyk-Swita et al., 2017)]. Intracellular chitosanases were also found in plants (Osswald et al., 1994). The biochemical properties of chitosanases depend on the source of enzymes, and their properties are summarized in Table 7. Most chitosanases are characterized by molecular masses, ranging from 10 to 75 kDa. However, many exceptions can be found in the literature; for example, chitosanase from *Aspergillus fumigatus* KH-94 has larger molecular weight of 108 kDa (Thadathil and Velappan, 2014). The optimum pH of microbial chitosanases' activity ranges from 4 to 8, while the optimum temperature varies from 30 to 60°C and is closely associated with the growth conditions of the microorganism that synthesizes them. Thermostable chitosanases have been reported in several articles (Chen X. et al., 2012; Zitouni et al., 2013; Doan et al., 2019a). Thermostability is particularly useful during enzymatic hydrolysis at higher temperatures, allowing chitosan to be dissolved at higher concentrations (Zitouni et al., 2013).

**Table 7:** The biochemical properties of chitosanases.

**Organism**	**pH optima**	**Temp. optima (^**°**^C)**	**Molecular mass (kDa)**	**Isoforms**	**Activation**	**Inhibition**	**Substrate specificity**	**References**
*Bacillus licheniformis* MB-2	6-7	70	75	–	Mn^2+^, Co^2+^, Ca^2+^, urea	Ni^2+^, Zn^2+^	Active against: chitosan with different DD; glycol chitosan, colloidal chitosan Inactive against: glycol chitin, colloidal chitin	Ekowati et al., 2006
*Paenibacillus* sp. 1794	4.8	80–85	40	–	ND	ND	Active against: chitosan, carboxymethylcellulose, chitosan or cellulose-derived hexasaccharides inactive against: chitin	Zitouni et al., 2013
*Mucor circinelloides*	5.5–6	37	42	–	Ca^2+^, Mn^2+^, Mg^2+^	Hg^2+^, Cu^2+^, Ag^2+^,	Active against: chitosan with high DD inactive against: colloidal chitin, sodium salt of carboxymethylcellulose, starch	Struszczyk et al., 2009
*Aspergillus* QD-2	5.6	55	ND	–	ND	ND	ND	Zhang et al., 2012
*Gongronella* sp. JG	5.6	55–60	28	–	Mn^2+^, Ca^2+^, Sr^2+^	EDTA-	Active against: colloidal chitosan inactive against: colloidal chitin, carboxymethylcellulose	Wang J. et al., 2008
*Serratia marcescens* TKU011	5	50	21	–	–	EDTA, Mn^2+^, Fe^2+^	Active against: chitosans with different DD inactive against: colloidal chitin and chitin	Wang S. et al., 2008
*Anabaena fertilissima*	7.5	27	ND	–	Cu^2+^, Zn^2+^	Ag^+^, Fe^3+^, Hg^2^	Active against: glycol chitosan, colloidal chitin, CM-chitosan, and colloidal chitosan (low)	Gupta et al., 2012
*Streptomyces roseolus*	5	50	41	–	Mg^2+^	Cu^2+^, Co^2+^Mn^2+^, Zn^2+^	Active against: colloidal chitosan, glycol chitosan (weakly), glycol chitin (weakly)	Jiang et al., 2012
*Bacillus cereus* D-11	6	60	41	–	–	Hg^2+^ Pb^2+^ Cu^2+^	Active against: colloidal chitosans very low activity against glycol chitosan, chitosan powder, chitin	Gao et al., 2008
*Acinetobacter calcoaceticus* TKU024	6	50	27	CHSA1	–	Mn^2+^	CHSA1 and CHSA2 active against: chitosans with different DD, chitin	Wang et al., 2011
*Acinetobacter calcoaceticus* TKU024	7	60	66	CHSA2	–	Mn^2+^		
*Staphylococcus capitis*	7	30	35	–	Mn^2+^ Zn^2+^, Cu^2+^	Ba^2+^, Mg^2+^, Ca^2+^ and Ni^2+^	Active against: soluble chitosan, colloidal chitosan, powdered chitosan inactive against colloidal chitin, carboxymethylcellulose	Sun et al., 2018

Based on their amino acid sequences, chitosanases are classified into seven families of glycoside hydrolases (GH-3, GH-5, GH-7, GH-8, GH-46, GH-75, and GH-80), further grouped into four classes based on their cleavage specificity (Weikert et al., 2017). GH-46, GH-75, and GH-80 contain only chitosanases, while the families GH-5, GH-7, and GH-8 contain other glycoside hydrolases, such as cellulase and xylanase (Viens et al., 2015). All known chitosanases can cleave GlcN-GlcN bond, while class I chitosanases can additionally cleave GlcNAc-GlcN bond. The best-known example of a class I chitosanase is an enzyme from *Streptomyces* sp. N174 which belongs to the GH-46 family. Another example of GH-46 chitosanase is an enzyme from *Bacillus* MH-K1, which was classified into class III. Enzymes belonging to this class are capable of cleaving GlcN-GlcNAc bond, in addition to GlcN-GlcN. Class II chitosanases, such as these from *Bacillus* sp. No. 7-M (GH-8), are limited to cleave GlcN-GlcN bonds exclusively. Another extensively studied and recently introduced a class of chitosanases is class IV belonging to the GH-46 family. Ando et al. (2008) revealed that chitosanases from this class could cleave all bonds except GlcNAc-GlcNAc. It was previously thought that chitosanases, in general, are not able to hydrolyse GlcNAc-GlcNAc bond. Surprisingly, results obtained by Heggset et al. (2010) abolished a clear distinction between chitinases and chitosanases. Strikingly, the chitosanase from *Streptomyces coelicolor* A3(2) was able to cleave all glycosidic bonds, including GlcNAc–GlcNAc. Until now, the members of the GH46 family have been the best characterized among all chitosanases. The chitosanases from GH46 family have a highly electronegative substrate-binding cleft, in contrast to other glycoside hydrolases of various substrate specificities (Viens et al., 2015). A high content of acidic residues (Asp and Glu) in the substrate-binding cleft is thought to be responsible for the high specificity of these proteins and their poor recognition of chitinous and highly N-acetylated substrates (Marcotte et al., 1996). Most chitosanases of the GH46 family are α-helical proteins, composed of two lobes which are separated by a substrate-binding cleft. The best-known enzyme from this family, in terms of structure and mechanism of hydrolysis of the β-1,4-glycosidic bond, is chitosanase isolated from the bacterial strain *Streptomyces* sp. N174. The molecular structure of this enzyme shows ten α-helices and three β-sheets. This chitosanase is dumbbell-shaped and 55 Å long containing two globular domains connected through a bent 27-residue backbone helix. The domain forms cleft, 10 Å wide and 12 Å deep. The binding of chitosan to the active site suggests that the mechanisms of substrate and catalytic binding may be similar to other glycohydrolases. The X-ray structure suggested that Glu 22 and Asp 40 are essential for the catalytic function of this chitosanase (Marcotte et al., 1996). The chitosanase from *Bacillus* MH-K1 has two globular upper and lower domains, which generate the active site cleft for the substrate binding. The molecular folding is similar to the described earlier chitosanase from *Streptomyces* sp. N174, but there is only 20% identity at the amino acid sequence level between both chitosanases. There are three regions of markedly different topology. The disulfide bridge between Cys50 and Cys124 joins the β1 strand and the α7 helix is unique to the structure and not conserved among other chitosanases. The orientation of two backbone helices, connecting the two domains, is also distinct. The helix is responsible for the differences in size and shape of the active site cleft in these two chitosanases. The discussed differences of the active site cleft are believed to be behind different substrate specificities of these enzymes. It has been proved that the size and shape of the cleft are such that the substrate sugar with the acetyl groups at positions suitable for the specific cleavage reaction can be accommodated in the active site, which affords reaction specificity for substrate recognition of this chitosanase (Saito et al., 1999).

**Figure 4:**
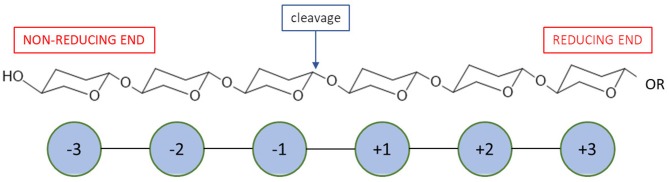
A schematic mode of action for enzymatic subsites of carbohydrate processing enzymes.

Most recent classification of chitosanases is based on the ability of enzymes to cleave bonds at GlcNAc residues positioned at a (−1) or (+1) subsite (Figure 4). It has been proven that this ability varies, especially when substrates have a different fraction of acylation. Weikert et al. (2017) showed that the current classification system is no longer tenable and might not be applicable to chitosanases. Conflicts with the recent classification are observed for the reactions in which high FA (fraction of acetylation) oligomers were used as substrates. They recommended a chitosanases classification system which is based on specificity and preferences toward subsite (−2) to (+2). Gercke et al. (2019) used rational protein engineering methods to produce modified chitosanase from *Bacillus* sp. The obtained enzyme was specific toward subsite (−3) to (+3) and able to produce DP4 COS by hydrolysing fully deacetylated substrates.

It has been reported that chitosanases from different sources may exhibit different catalytic mechanisms dependent, among other factors, on the DD of substrates (Sinha et al., 2016). Moreover, microbial chitosanases produce a relatively higher yield of COS, comparing to enzymes from other sources (Kim and Rajapakse, 2005). Kohlhoff et al. (2017) characterized chitosanase from *Alternaria alternate*. The enzyme showed a specific cleavage pattern exclusively toward bonds following GlcN-GlcNAc pairs. Moreover, the optimum activity was observed against moderately acetylated chitosans, low activity against fully acetylated or fully deacetylated chitosans, and no activity against glycol chitin. Qin et al. (2018) conducted a controllable preparation of COS in mild conditions (30°C, pH 5.5) using a novel cold-adapted chitosanase from a Rhizobacterium *Gynuella sunshinyii*. With chitosan as a substrate, the main products obtained were (GlcNAc)_2_ and (GlcNAc)_3_ (yield = 94.6%). The enzyme belongs to the GH-46 family and showed no catalytic specificity toward chitin. Studies on chitosanase produced by *B. subtilis* conducted by Su et al. (2017) showed a promising ability of this enzyme to hydrolyse chitosan to (GlcNAc)_2_, (GlcNAc)_3_ and (GlcNAc)_4_. Nidheesh et al. (2015) investigated chitooligomers production by crude chitosanase from *Purpureocillium lilacinum* CFRNT12. The enzyme was tested on different chitosan substrates (colloidal and crystalline chitosan). For both substrate forms, the maximum concentration of chitooligomers was observed after 24 h of hydrolysis. The enzyme performed the endo- type of hydrolysis. Chitosan trimers and tetramers accounted for the majority of the product.

The efficiency of traditional glycoside hydrolases (GHs), such as chitinases and chitosanases, is highly limited due to the strong crystallinity and insolubility of chitin in an aqueous environment. To tackle this problem, pre-treatment of native chitin is necessary for efficient depolymerization of the substrate. Traditionally, chitin is treated with strong acids, such as hydrochloric acid (for colloidal chitin) (Guo et al., 2017) or phosphoric acid (for swollen chitin) (Bansode and Bajekal, 2006), to maximize its enzymatic accessibility. In contrast to conventional GHs, Lytic Polysaccharide Monooxygenases (LPMOs) are capable of directly cleaving glycolic bonds in highly crystallized chitin, increasing its accessibility for subsequent enzymatic processing (Eijsink et al., 2019).

**LPMOs** (EC 1.14.99.53-56) are enzymes capable of cleaving glycolic bond in crystalline polysaccharides through oxidizing either C1 or C4 of the glucopyranose ring. The oxidation mechanism requires a reductant (such as ascorbic acid), an oxygen-containing co-substrate (O_2_ or H_2_O_2_), and a single bound copper ion. LPMOs share a typical immunoglobulin-like β-sandwich core structure, and most LPMOs are single domain enzymes (Vaaje-Kolstad et al., 2017). LPMOs have been assigned to auxiliary activity (AA) families AA9, AA10, AA11, AA13, AA14, and AA15 in the Carbohydrate-Active Enzymes (CAZy) database (Tandrup et al., 2018). These enzymes act on a range of polysaccharides including cellulose, chitin, starch, xyloglucan, glucomannan, and cellodextrins. Chitin-active LPMO was first demonstrated in 2010 for the *Serratia marcescens* AA10 (CBP21) (Vaaje-Kolstad et al., 2010). To date, LPMOs with chitinolytic activity has been expanded to families AA10, AA11, and AA15 (Hemsworth et al., 2015). The majority of chitinolytic LPMOs reported to date have been observed in fungi and bacteria in organisms such as *Streptomyces gresius, Enterococcus faecalis*, and *Bacillus thuringiensis*. The presence of chitinolytic LPMO genes has also been reported in a virus (Chiu et al., 2015) and even an arthropod (Sabbadin et al., 2018). Bai et al. reported that chitinolytic AA10 LPMO genes are present in about one-third of terrestrial bacterial genomes but absent in strict anaerobes (Bai et al., 2016).

Additionally, when combined with GHs, LPMOs contribute synergistically to overall substrate solubilization and thus significantly reduce the load of enzyme cocktails (Mutahir et al., 2018). Nakagawa et al. (2013) studied the enzymatic depolymerization of α-chitin with varying particle size and crystallinity produced by mechanical pre-treatment. It was found that the synergies between LPMO (CBP21 from *S. marcescens*) and monocomponent chitinases were clear for all substrates but more significant for substrates with high degrees of crystallinity. Hamre et al. (2015) reported that LMPO (CBP21) could boost the apparent k_cat_ values of exo-active chitinases ChiA and ChiB, of which the apparent k_cat_ values were boosted from 1.7 ^1^ and 1.7 s^−1^ to 11.1 and 13.9 s^−1^, respectively. However, such effect was not observed in endo-active chitinase ChiC. Mekasha et al. (2017) optimized the proportion of chitinases (*Sm*ChiA, *Sm*ChiB, *Sm*ChiC), an LMPO (*Sm*LPMO10A) and a beta-N-acetylhexosaminidase (*Sm*CHB) for the saccharification of shrimp and crab chitins. It was found that when *Sm*LPMO10A was present at 3 and 2%, for shrimp and crab chitin, respectively, the corresponding saccharification yields reached 70%-75%. These results were significantly higher than those of a “minimal” cocktail of *Sm*ChiA and *Sm*CHB where only 40% yield of saccharification was achieved. LPMOs could also be used to prepare functionalized chitin materials. Wang et al. (2018) successfully introduced 35 nmol of carboxylate (COO^−^) moieties per milligram of α-chitin with a new LPMO *Ff* AA11, without affecting the crystallinity of the chitin fibers. In the same research, LPMO in combination with a chemical method was also employed to transform recalcitrant chitins into desirable functionalized (nano)materials.

## Biological Activity of Chitin and its Derivatives

The specific properties of chitin provide numerous potential applications of this biopolymer. Unfortunately, the use of chitin is significantly limited due to the low reactivity and lack of solubility in water and common organic solvents. The most useful chitin derivative is chitosan, which is beneficial for biomedical applications due to biocompatibility, biodegradability and low toxicity. The most important biological activities of chitosan and its degradation products (COS) include antimicrobial, antiviral, antitumor, and antioxidant activities. The spectrum of antimicrobial activity of chitosan and COS includes bacteria, filamentous fungi, and yeast. Chitosan, however, shows its antimicrobial activity only in an acidic medium because of its poor solubility above pH 6.5. Thus, water-soluble COS may be good candidates as a polycationic biocide. The mechanism of their antimicrobial activity has not yet been clearly explained. According to Liaqat and Eltem (2018) contradictions in the proposed mechanisms may be the result of the use of various microorganisms and methods in research, as well as the quality, purity and characteristics of the COS being analyzed. One of the theories explaining this mechanism says that the inhibitory effect of chitosan and COS on bacterial growth is related to their polycationic nature, resulting from the presence of free -NH_2_ groups in units of D-glucosamine forming the chains of these compounds. This enables them to bind strongly to carboxyl groups with negative charge of compounds building external cell membranes of microorganisms (Kittur et al., 2003; Vishu Kumar et al., 2005, 2007). Chitosan and its oligomers can reduce the permeability of the cell membrane, forming a coating on its surface and thereby blocking cell access to external nutrients, which leads to its death (Vishu Kumar et al., 2007). It is generally recognized that the number of -NH_2_ groups and also the antibacterial activity often increases with the simultaneous increase of their DP value (Vishu Kumar et al., 2005). The higher activity of chitosan degradation products in relation to the high molecular biopolymer is explained by the possibility of the former penetrating the cells, where they block RNA transcription as a result of adsorption with bacterial DNA (Kim et al., 2003; Mei et al., 2015). The mechanism of interaction of chitosan and its degradation products with bacterial cells depends to a large extent on the structure of the cell wall of a given microorganism. In the case of gram-positive bacteria having a cytoplasmic membrane covered with a cell wall formed of several dozen layers of peptidoglycan containing negative GlcNAc, N-acetylmuramic acid, numerous amino acids, or teichoic acids, primarily for strong binding characterized by the opposite charge COS and LMWCh. This causes deformation of the bacterial cell wall, which in turn is associated with the exposure of the cytoplasmic membrane to osmotic shock, the burst of the cytoplasm and ultimately the death of bacteria. In contrast, the gram-negative bacterial cell contains an outer membrane consisting, among others from lipopolysaccharides (LPS) and proteins; a cell wall with only 1–3 layers of peptidoglycan and a cytoplasmic membrane. Negatively charged O-specific side polysaccharide chains form an ionic type combination with COS or LMWCh amine groups. In the case of COS, cell access to external nutrients is blocked. Due to the strong binding of LPS side chains to the outer membrane of the cell, its destruction does not occur—as was the case with the gram-positive group of bacteria. The smaller the DP of chitosan degradation products and the higher the electronegative charge of bacteria, the easier the associated and aggregation of these compounds occurs, and thus the blockade of the supply of external nutrients and the final cell death (Vishu Kumar et al., 2005). On the other hand, the charge of oligomers with a higher DP, i.e., LMWCh, is large enough to remove the LPS associated with them from the cell membrane and subsequently to cell lysis (mechanism as in the case of gram-positive bacteria) (Vishu Kumar et al., 2007).

The antimicrobial properties of chitosan and its degradation products depend on many factors, including their source and concentration, molecular weight and deacetylation degree, and the strain of the microorganism on which they were tested (Kyoon et al., 2003; Liu et al., 2006; Li et al., 2014; Laokuldilok et al., 2017; Bonilla et al., 2019; Shi et al., 2019). It was found that in the case of COS, their DP with a value of not less than five is essential for antibacterial activity of fully deacetylated COS (Li et al., 2014). Jeon et al. (2001) indicated that COS exhibits antimicrobial activity against Gram-positive and Gram-negative bacteria. However, high-molecular-weight COSs (5 000–10 000 Da) exhibited higher antimicrobial activity than low-molecular-weight COSs. It has been proven that positively charged COSs interact with negatively charged bacterial cell walls, resulting in suppression of the metabolic activity of bacteria by reducing nutrient permeation through the cell wall. Therefore, the death rate of bacterial cells increases upon an increase in the DD of COSs (Tsai et al., 2002). On the other hand, reports are confirming that acetylated sequences in COS structure are essential for their antimicrobial activities, and COS comprising more number of acetylated sequences (less number of free amino groups) have shown higher antimicrobial activities (Sánchez et al., 2017). Further work is needed to determine the mechanism of antimicrobial activity of chitosans and COS and to affect their activity primarily DD and DP. Examples of antimicrobial activities of chitosan and chitooligosaccharides are summarized in Table 8. The antifungal activity of chitosan is commonly used in agriculture for the reduction of mycelial growth, sporangial production, release of zoospores, germination of cysts and the induction of local and systemic resistance (Atia et al., 2005) Additionally, results reported by Mei et al. (2015) proved the potential of COS for clinical application. Enzymatically produced, well-characterized chitooligosaccharides exhibited excellent antifungal properties against dermatophyte fungus *Trichophyton rubrum* in a guinea pig model.

**Table 8:** The antimicrobial activities of chitosan and its degradation products.

**Chitosan/COS**		**Activity against**	**References**
**MW [kDa]/DP**	**DD [%]**		
MW 1–10	75	*Vibrio parahaemolyticus*	Park et al., 2004
MW 8; 66; 197	85	*E. coli*, *S. aureus*, *Candida albicans*, *C. tropicaliss*	Zhang et al., 2019
DP 2–12	–	*Alternaria alternate, Rhizopus stolo* *Botrytis cinereanifera*	Oliveira et al., 2008
MW 49.5; 138 and 142	91	*E. coli*, *S. aureus*, *C. albicans*	Pan et al., 2019
MW3 0–10; 10–5; <5	84	*E. coli*, *Listeria monocytogenes*	Sánchez et al., 2017
MW 5.1; 14.3 and 41.1	99	*E. coli*, *Salmonella typhimurium, Salmonella enteritidis*	Laokuldilok et al., 2017
MW 194		*Staphylococcus aureu*	
MW 28	89	*S. typhimurium*	Jeon et al., 2001

There are several reports on the antiviral properties of chitosan and COS, but the mechanism of their activity has also not yet been clearly explained. Chitosan, as well as its degradation products, most likely inhibit viral infections by reducing virus infectivity and inducing the resistance of plant and animal organisms. Suppression of infectivity may also be associated with preventing the absorption of viral particles into the cell membrane. The sulphated COS with MW in the range of 3–5 kDa is an effective compound to stop replication of HIV-1 virus by blocking viral entry and virus-cell fusion probably via disrupting the binding of HIV-1 to CD4 cell surface receptor (Artan et al., 2009). The study of antiviral activity of chitosan oligomers with MW from 17 to 2 kDa and DD 98.5, 83, and 75% were tested against the tobacco mosaic virus by Davydova et al. (2011). The obtained results confirmed that these samples inhibited the formation of local necrosis induced by the virus by 50–90%.

Chitosan and COS-like chitosans can be considered as potential anticancer agents because of their anti-tumor activities. Unfortunately, the mechanism of their action on tumor cells has not been elucidated to date. Huang et al. (2006) proposed a hypothesis according to which COS as a negatively charged polysaccharides that can adsorb on a cancer cell. The electrostatic interactions between cancer cells and polycationic polymer significantly change the permeability of cancer cells. Mattaveewong et al. (2016) suggest that tumor cells are not killed directly by COS. These small oligosaccharides suppress the NF-κB and mechanistic target of rapamycin (mTOR) by AMP-Activated Protein Kinase (AMPK) activation. Recent research revealed the potential of COS as an immunostimulatory agent which may be used in anticancer therapies related to immunomodulation (Zheng et al., 2016; Xing et al., 2017). The molecular weight of COS has an essential effect on anticancer activity. It has been reported that chitohexanoses are the most promising oligomers to manifest the anticancer effect (Xiong et al., 2009; Li et al., 2011). Wang et al. (2007) published the results of studies confirming the influence of the degree of COS acetylation on anticancer activity. The antiangiogenic activity of acetylated COSs was significantly stronger than the parent oligosaccharide. Other research indicated that antiangiogenic activity of COS is also dependent on FA and DP of oligomers and that the FA is more critical of the two parameters (Wu et al., 2012). Chitosan and its derivatives were used as transporters of anti-cancer drugs. It has been investigated that anticancer agents conjugated with chitosan can execute anticancer effects with a decrease of side effects and gradual release of free drug in the cancer tissues (De Campos et al., 2001; Janes et al., 2001). Liposome-chitosan nanoparticles were used to obtain dose-dependent tumor-weight inhibition drug release system, which showed promising results in *in vivo* studies (Li et al., 2009). Yin et al. (2017) reported that the COS (MW 2,000–5,000 Da) tethered on the liposomes through disulphide linkers (-SS-) to cholesterol may be an excellent platform for cytoplasmic delivery of anticancer drugs. An amphiphilic all-trans-retinoic acid (ATRA) conjugated COS nanoparticles also revealed the promising potential as drug carriers for co-delivery of ATRA, paclitaxel, and other hydrophobic therapeutic agents (Zhang J. et al., 2015).

In recent years, the possibility of using chitosan and COS as free radical scavengers are also of significant interest. It is known that the mechanism of their antioxidant activity is associated with the presence of free amino group in the glucopyranose rings, which by reacting with free radicals form stable forms of macro-radicals. In addition, the -NH_2_ groups exhibit chelating properties concerning many metal ions, including Fe^2+^, which are activators in the formation of hydroxyl radicals—the most dangerous for the human body. Antioxidant activities of chitosan and COS are affected by DD and MW (Park et al., 2004; Zhao et al., 2013). Studies by Park et al. (2004) suggested that the scavenging activity of chitosan depended on its DD and chitosan with a higher DD exhibited better scavenging activity. In contrast, chitosan oligosaccharides (MW 5 kDa, DD 97%) and its derivatives tested by Zhao et al. (2013) showed a higher scavenging effect than chitosan used to obtain them (MW 120 kDa, DD 97%). Like other properties of chitooligosaccharides, their antioxidant activity is also dependent on the physicochemical properties of COS. Studies attempted to determine the relationship between antioxidant activity of COS and their MW indicated that that low MW (5,000 Da) COS had shown the highest antioxidant capabilities. Additionally, it has been found that the antioxidant activity of COS can be predicted based on the composition of oligomers expressed as the ratio of acetylated vs. deacetylated units (Mengíbar et al., 2013). Antioxidant activity of COS is another promising characteristic which can be used to produce value-added products for food preservation and functional food. Studies conducted by Yang et al. (2017) play an active part in the prevention of beer flavor deterioration by inhibiting the formation of staling compounds and increasing radical scavenging activity. The activity of COS was dependent on the molecular weight of oligomers. Additionally, COS showed radical scavenging activity in the finished beer, which is expected to improve the shelf life stability during beer storage.

The biodegradability of chitin and chitosan was principally attributed to their susceptibility to enzymatic hydrolysis by lysozyme, that exists in all human body tissues. It has been demonstrated that chitosan can also be metabolized in animal and human tissues by the combined action of lipase and chitosanases (Poshina et al., 2018). Thus, chitosan and its derivatives have been considered as promising vehicles for oral prolonged-release drugs and as a matrix in drug release systems in the form of beads and granules. Physical hydrogels of chitosan which are usually used for this purpose can be formed by various reversible links such as ionic interactions (crosslinked hydrogels) and polyelectrolyte complexes (PEC), or secondary interactions (chitosan/poly(vinyl alcohol) complexed hydrogels), grafted chitosan hydrogels, and entangled hydrogels (Berger et al., 2004). PECs of chitosan with polyanions of natural origin like pectin, alginate, carboxymethyl cellulose, or with synthetic ones like poly (acrylic acid) have been discovered as matrices for controlled-release systems (Berger et al., 2004). Chandy et al. (2002) reported that chitosan-polyethene glycol-alginate microspheres are suitable materials for the delivery of low molecular weight (LMW) heparin with antithrombotic properties. Chitosan and its derivatives can be used to form products with haemostatic properties. It has been found that in the initial phase of chitosan/blood interactions, plasma proteins absorb on chitosan-based systems. In the next step, the adhesion and activation of platelets occur, which leads to the formation of a thrombus (Yeh and Lin, 2008). It was claimed that chitosan was hypocholesterolemic and hypolipidemic (Domard and Domard, 2002). Pan et al. (2016) investigated that functional food based on the chitosan and its derivatives effectively improve liver lipids metabolism and protect the liver from the oxidized trauma by enhancing hepatic function. Biocompatible, natural and synthetic carriers are commonly used in tissue engineering techniques as a support for initial cell attachment and subsequent tissue formation. Chitosan shows a similar spatial structure as glycosaminoglycans (GAGs) found in the extracellular matrix of several human tissues. The physical and chemical properties of chitosan facilitate the adhesion of the cells and maintenance of the differentiating functions (Croisier and Jérôme, 2013). Gelatin-chitosan hydrogels were successfully used as a culture substratum for respiratory epithelial cells. However, two-dimensional gel conformation was not sufficient to induce very high ciliogenesis and mucus secretion (Risbud et al., 2001). A three-dimensional biodegradable hydroxyapatite/chitosan-gelatin network was used as a biomimetic scaffold for bone cells growth and proliferation. The obtained cell/scaffold constructs had good biomineralization effect after 3 weeks in culture (Zhao et al., 2002). As a polycationic biopolymer, chitosan and its derivatives can form complexes with nucleic acids. This property was utilized in gene transfection experiments, in which chitosan with DD around 80–90% has proved useful as a gene carrier for *in vitro* and *in vivo* processes (Köping-Höggård et al., 2001; Mao et al., 2001; Kwon et al., 2013). It has been demonstrated that the reduction of chitosan DD results in a reduction of DNA binding efficiency and consequently in a decreased expression of transfected genes (Kiang et al., 2004; Huang et al., 2005). Furthermore, complexes formed with higher molecular weight chitosan are more stable and demonstrate higher transfection efficiency (Bordi et al., 2014). In addition to the indicated examples of chitosan applications, this biopolymer has been used in many other industries, e.g., as adsorbents for dye removal from water and wastewater (Vakili et al., 2014), as ingredients of cosmetic that increases the water-resistance of emulsions protecting against sun irradiation and consequently enhances its film-forming ability (Aranaz et al., 2018), as a food ingredient (Shahidi et al., 1999), as a carrier for enzyme immobilization (Biró et al., 2008; Hou et al., 2019).

As it was mentioned, conventional methods of chitosan and chitooligosaccharides preparation are difficult to control and often lead to a mixture of products with different properties. The indicated examples clearly show that the biological activities of COS are significantly affected by the DA, DP, MW, FA, and PA; therefore it is crucial to develop fully controlled production methods of chitosan and chitooligosaccharides—application of appropriate enzymes (biocatalysts) can be very helpful in achieving this goal.

## Enzymatic Transformations of Chitin/Chitosan—What the Future Holds?

The disadvantages of the currently used industrial methods of chitosan manufacturing and the increasing demand for a broad range of novel chitosan oligosaccharides with a fully defined architecture attract growing interest in the chitin- and chitosanolytic enzymes. Due to their unique abilities, these enzymes are increasingly seen as a useful tool toward biotechnological chitosan and COS production, especially when a controlled non-degradative and well-defined process is required.

As previously mentioned, enzymatic modification of chitin includes deacetylation of chitin into chitosan and depolymerization of chitin or chitosan into acetylated or deacetylated chitooligosaccharides, respectively. Unfortunately, the chemical structure and highly crystalline character of native chitin seem to limit the accessibility of the enzyme to reactive polymer groups. The solution of the problem may be the application of multiple chitin- and chitosanolytic enzyme cocktails or complexes enabling comprehensive modification of the native substrate. Recent research indicated that the chitinases of microbial origin could depolymerise and thus potentially to loosen the crystalline chitin structure (Guo et al., 2017). Considerable interest emerged by the discovery of chitin active lytic polysaccharide monooxygenases (LPMOs), which are capable of directly cleaving glycolic bonds in highly crystalline chitin (Mutahir et al., 2018). Moreover, it was found that the synergies between LPMO (CBP21 from *S. marcescens*) and monocomponent chitinases were clear for all substrates but more significant for substrates with high degrees of crystallinity (Mekasha et al., 2017). Accordingly, the pre-action of enzymes capable of fragmenting the chitin chain can significantly increase the susceptibility of the intermediates to the action of chitosanases, chitin deacetylases, and chitooligosaccharides deacetylases. The joint action of sequential enzymes in multiple-enzyme cocktails or complexes yield efficient transfer of an intermediate from one enzyme to the next enzyme, thereby resulting in an enhanced reaction rate. Simultaneous use of chitin- and chitosanolytic enzymes involved in chitin and chitosan modification can significantly reduce the diffusional length of the intermediates along the multi-reaction pathway. High substrate specificity of enzymes involved in the multienzymatic mixture, potentially allows obtaining products with strictly defined chain arrangement, and thus desirable biological properties. The proposed solution can eliminate the problems associated with the relatively low efficiency of modification of native chitin by currently known enzymes.

The multi-step character of enzymatic chitin modification processes and high costs of enzymes preparations are other limiting factors enabling the application of enzymatic route on a wide range. Substantial improvement in the cost-effectiveness of enzymatic processes can be obtained by the assembly of numerous enzymes and co-enzymes *in vitro* in so-called cascade biocatalysis (You et al., 2012; Liu et al., 2013). Expression of fusion proteins with multifunctional activity can also significantly reduce the cost of enzymatic methods (Iturrate et al., 2009) and provide desirable binding properties toward the chitin (Hou et al., 2019). However, it is sometimes challenging to obtain multifunctional proteins that keep the activity and substrate specify of native enzymes. Another strategy involves a co-expression of genes coding enzymes required for multi-step processes. By simultaneously producing several enzymes, it is possible to significantly reduce the costs of the process in which the use of several biocatalysts is required. Commonly, a multi-gene expression is based on constructs harboring the pathway genes under the separate control of the same or different promoters and terminators. Unfortunately, the repeated use of homologous sequences typically results in genetic instability. On the other hand, the use of different regulatory sequences results in the expression of individual genes with different efficiencies (Geier et al., 2015). The multi-gene expression from single, polycistronic transcript can be used to reduce the number of regulatory elements. Since eukaryotes generally do not express polycistronic operons, other regulatory elements are needed to initiate the multi-gene expression. One way to achieve polycistronic expression is the use of internal ribosome entry sites (IRES). The IRES serves as a launching pad for the internal initiation of translation. The approach allows expression of two or more genes from a single transcript. However, these sequences are relatively large (~500 bp) which significantly affects the size of constructs used for transformation, thereby lowering the efficiency of the process. Moreover, the application of IRES in polycistronic expression results in as much as a 10-fold lower expression of the downstream coded protein (Douin et al., 2004; Ha et al., 2010). Another approach involves the use of viral self-processing 2A sequences. These short oligopeptides (around 20 aa) responsible for the phenomenon of ribosomal skipping (Donnelly et al., 2001) have been identified in several members of the picornavirus family, where they participate in a self-cleavage of viral polypeptides to generate the mature viral proteins. These sequences have been successfully used for the production of therapeutic proteins, antibodies, vaccines and in gene therapy, both in transgenic mice and animals cells (Ha et al., 2010). The ribosomal skipping activity of 2A sequences has been implemented for polycistronic expression of proteins in transgenic plants (Penn, 2000) and yeasts (Geier et al., 2015).

Another exciting alternative that can increase the efficiency of enzymatic methods for the production of chitin derivatives is the creation of multi-enzymatic complexes that allow the cascade effect of biocatalysts. Most naturally occurred cascade enzymes in metabolic pathways are spatially held together by non-covalent protein-protein interactions (Srere, 2002). This makes the active sites of each enzyme closer together triggering the phenomenon of substrate channeling, wherein an intermediary metabolic product of one enzyme is passing directly to another active site (another enzyme) without its release into the reaction medium. Wilner et al. (2009) obtained even 30 fold increase in reaction efficiency by linking horse peroxidase and glucose oxidase by DNA scaffolds of different length. Moehlenbrock et al. (2010) significantly improved the current and power density of biofuel cells through the chemical, covalent linkage of proteins using the mitochondria isolated from *Saccharomyces cerevisiae*. Kim and Hahn (2014) synthesized cascade-enzymes scaffold system applied to the improved production of 2,3-butanediol. The functioning of the system was based on cohesin-dockerin interactions, which allowed to increase the production titer up to 37%. The current state of knowledge and modern methods of genetic engineering, molecular biology and related sciences give a real chance to develop an efficient and controlled method of enzymatic modification of chitin and its derivatives.

## Author Contributions

MK devised the topic, collected most of the data, participated in preparation of draft manuscript, and participated in assembly and editing of the final manuscript. KS-S and XL collected data and participated in preparation of draft manuscript. MS-A participated in preparation draft manuscript. MD devised the topic participated in preparation of draft manuscript and participated in assembly and editing of the final manuscript.

### Conflict of Interest

The authors declare that the research was conducted in the absence of any commercial or financial relationships that could be construed as a potential conflict of interest.

## Abbreviations

COS: chitooligosaccharides
CAGR: Compound Annual Growth Rate
ChDa: chitin deacetylase
CODa: chitooligosaccharides deacetylase
LPMOs: lytic polysaccharide monooxygenases
GHs: glycoside hydrolases
GlcNAc, 2-acetamide-2-deoxy-D-glucopyranose: N-acetyl-D-glucosamine
GlcN, 2-amino-2-deoxy-D-glucopyranose: D-glucosamine
DA: degree of acetylation
MW: molecular weight
DP: degree polymerization
DD: degree of deacetylation
DMF: dimethylformamide
DMSO: Dimethyl Sulfoxide
FA: fraction of acetylation
PA: pattern of acetylation
LDL: low-density lipoproteins
CE4: carbohydrate esterase family 4
CAZY: Carbohydrate-Active Enzymes Database
WSCT-50: water-soluble chitin with DD 50%
ACE: angiotensin-converting enzyme
TLC: thin-layer chromatography
GH-18, GH-19, GH-20: glycosyl hydrolase families
LMWCh: low molecular weight chitosan
LPS: lipopolysaccharides
GAGs: glycosaminoglycans
NF-κB: nuclear factor κB
mTOR: mechanistic target of rapamycin
AMPK: AMP-Activated Protein Kinase
ATRA: all-trans-retinoic acid
PEC: polyelectrolyte complexes
LMW: low molecular weight
IRES: internal ribosome entry site.
